# Weighted gene coexpression network analysis identifies the key role associated with acute coronary syndrome

**DOI:** 10.18632/aging.103859

**Published:** 2020-10-14

**Authors:** Yong Wang, Liu Miao, Lin Tao, Jian-Hong Chen, Chuan-Meng Zhu, Ye Li, Bin Qi, Fei Liao, Rong-Shan Li

**Affiliations:** 1 Departments of Cardiology, Liuzhou People’s Hospital, Liuzhou 545006, Guangxi, People’s Republic of China

**Keywords:** acute coronary syndrome, weighted gene coexpression networks, function enrichment, validation, immune inflammation

## Abstract

The present study sought to identify potential hub genes and pathways of acute coronary syndrome (ACS). We downloaded the dataset (GSE56045) from the Gene Expression Omnibus (GEO) database and analyzed weighted gene coexpression networks (WGCNA). Gene Ontology annotation, Disease Ontology and Kyoto Encyclopedia of Genes and Genomes (KEGG) pathway enrichment analyses were performed using R software. The protein-protein interaction (PPI) network was constructed using Cytoscape, and the Molecular Complex Detection app was employed to identify significant modules and hub genes. The hub genes were then validated in other microarrays and patients by RT–PCR. Two modules were identified and associated with coronary artery disease (CAD) and included 219 genes. After function and PPI analyses, 24 genes were identified to be potentially associated with CAD. Linear correlation was performed to calculate the relationship between the gene expression levels and coronary artery calcification score and found that *CCR7* (R = -0.081, *P* = 0.0065), *CD2* (R = -0.075, *P* = 0.0012), *CXCR5* (R = -0.065, *P* = 0.029) and *IL7R* (R = -0.06, *P* = 0.043) should be validated in other dataset. By comparing the gene expression levels in different groups in GSE23561, GSE34822, GSE59867, GSE60993 and GSE129935, only two genes (*CCR7* and *CXCR5*) showed significance. The nomogram showed that *CXCR5* showed the risk of ACS. Further analysis in chest patients found *CXCR5* played a key role resulting in ACS. Our WGCNA analysis identified *CXCR5* as a risk factor for ACS, and the potential pathogenesis may be associated with immune inflammation.

## INTRODUCTION

Coronary artery disease (CAD), according to incomplete statistics from the World Health Organization (WHO), still shows the highest incidence rate and mortality rate. With the improvement of living conditions, the incidence rate will continue to increase [1]. Many causes can lead to CAD. The most common reasons are uncontrolled blood pressure and serum, and an unhealthy lifestyle, such as smoking, drinking, mental stress and lack of sleep [2, 3]. The essence of CAD is coronary atherosclerosis. With the development of research technology, more studies have shown that atherosclerosis is a chronic inflammatory process [4]. Therefore, exploring the molecular mechanism related to coronary atherosclerosis may identify a very effective way to treat CAD.

During inflammation, the most obvious change in blood components is the sharp increase in the total number of white blood cells. Lymphocytes, the smallest type of white blood cell (WBC), is produced by lymphoid organs and is an important cell component of the immune response function of the body. Lymphocytes have immune recognition function and can be divided into T lymphocytes (also known as T cells), B lymphocytes (also known as B cells) and natural killer (NK) cells according to their migration, surface molecules and function [5]. Exploring the molecular mechanism of lymphocyte-mediated immune inflammation is becoming an important link in the prevention and treatment of CAD [6].

Advances in technology have led to a better understanding of the molecular mechanisms of disease onset. In the face of more gene sequencing data, choosing the most suitable analysis method is helpful [7], and weighted gene coexpression network analysis (WGCNA) can select the most directly related genes [8]. Presently, we detected the mRNA expression profile of WBC samples to identify highly connected hub genes and significant modules to show the potential molecular mechanisms.

## RESULTS

### Data preprocessing

We obtained 47,280 probes from each sample expression profile in GSE56045. After data preprocessing, we obtained 20,918 probes containing gene symbols from 1,202 samples. The gene expression matrix was associated with the sample phenotype matrix for further analysis.

### Weighted gene coexpression networks

**Figure 1:**
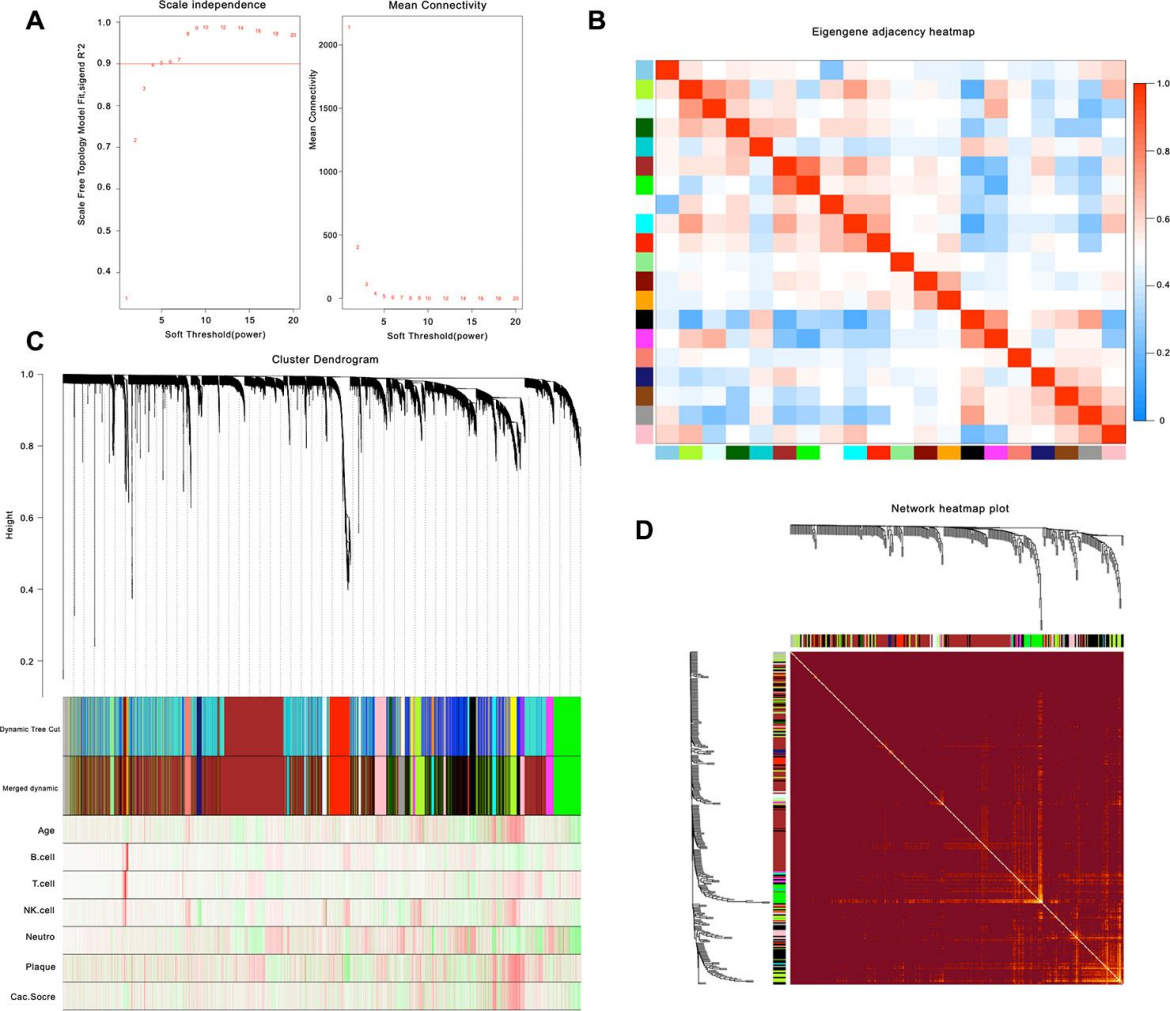
**Weighted gene co-expression network analysis.** (**A**) Analysis of network topology for various soft-thresholding powers. (**B**) Relationship among all the modules. (**C**) Clustering dendrogram of genes. Gene clustering tree (dendrogram) obtained by hierarchical clustering of adjacency-based dissimilarity. (**D**) Heatmap of the topological overlap in the gene network.

After calculation, we believe that, when the correlation coefficient is 0.9 (soft threshold β is 4), the coexpression network has a higher correlation and is more suitable to construct different gene modules (Figure 1A). Together with the topological overlap matrix (TOM), we performed the hierarchical average linkage clustering method to identify the gene modules of each gene network (deepsplit = 2, cut height = 0.4) and then showed the heatmap in Figure 1B. Next, we calculated the gene cluster tree and showed the results in Figure 1D. Finally, about eleven gene modules should be handled by the dynamic tree cut (Figure 1C).

### Interest module and functional annotation

**Figure 2:**
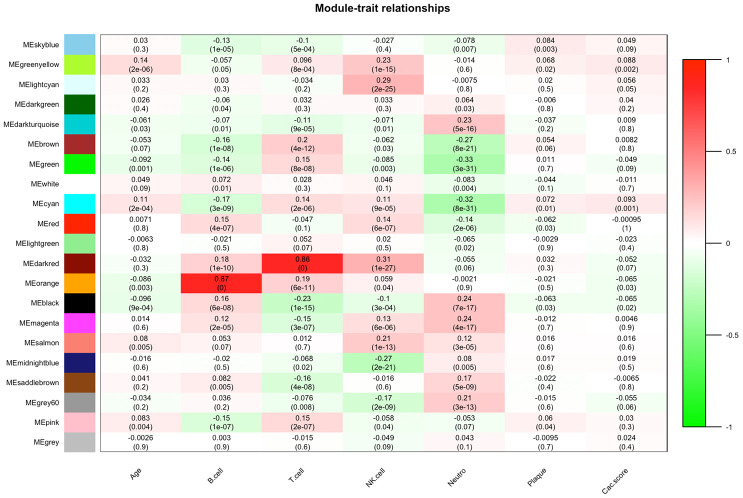
**Module-feature associations.** Each row corresponds to a module Eigengene, and each column to a clinical feature. Each cell contains the corresponding correlation in the first line and P-value in the second line. The table is color coded by correlation according to the color legend.

**Figure 3:**
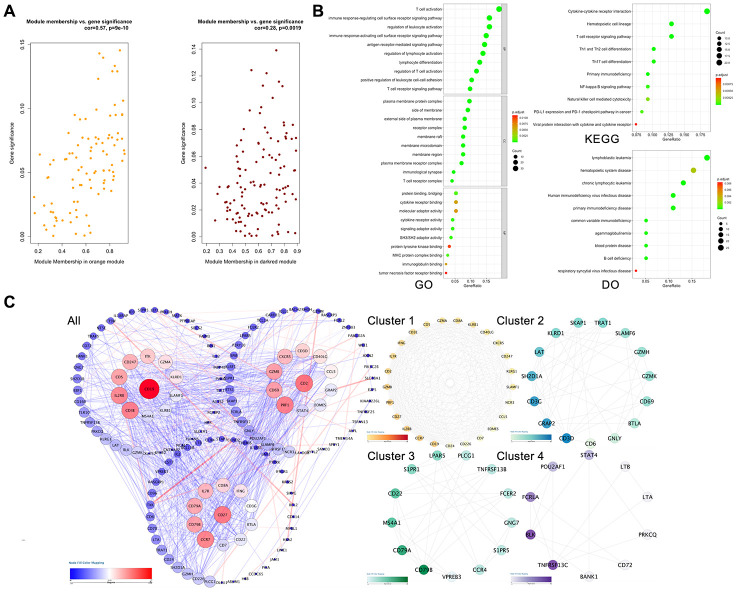
**Hub gene screening process.** (**A**) Association with gene significance and modules. The correlation between the module traits and the significance of gene expression. The higher the correlation, the higher the correlation between the gene expression in the module and the module trait. (**B**) Function annotation: (GO) Gene Ontology; (KEGG) Kyoto Encyclopedia of Genes and Genomes pathway enrichment; (DO) Disease Ontology; (**C**) Protein-protein interaction network of the selected genes. Edge stands for the interaction between two genes. A degree was used for describing the importance of protein nodes in the network, red shows a high degree and blue presents a low degree. The depth of the color represents the degree of correlation, and the deeper the color, the higher the correlation. The significant modules identified from the PPI using the molecular complex detection method with a score of >6.0. All: all of the genes; Cluster 1: MCODE-1= 12.07; Cluster 2: MCODE-2 = 9.92; Cluster 3: MOCDE-3 = 7.39; Cluster 4: MCODE-4 = 6.28.

As shown in Figure 2, the highest association in the module-feature relationship was the dark-red module and T cell (*r^2^* = 0.86, *P* < 0.001), orange module and B cell (*r^2^* = 0.87; *P* < 0.001). The dark-red module contains 121 genes, while the orange module contains 98 genes. All of these genes were showed in Supplementary Table 1. To determine the correlation between gene significance and color module, we conducted an in-depth calculation. As shown in Figure 3A, the correlation between the gene significance and orange module was 0.57 (*P* = 9E-10) and that of the dark red module was 0.28 (*P* = 0.0019). After confirming the correlation between the gene significance and modules, we analyzed the functional enrichment of the 219 genes in these two modules. Gene Ontology (GO) function, KEGG pathway enrichment and Disease Ontology analyses were performed by R (Figure 3B). The details of these analyses can be found in Supplementary Table 2. The biological processes of these two modules were found to be associated with GO:0042110-T cell activation (*P* = 4.89E-19), GO:0002768-immune response-regulating cell surface receptor signaling pathway (*P* = 4.66E-14), GO:0042113-B cell activation (*P* = 8.13E-07), and GO:0002699-positive regulation of immune effector process (*P* = 7.25E-05). However, in KEGG pathway analysis, these two modules were found to be associated with hsa04660-T cell receptor signaling pathway (*P* = 4.54E-09), hsa04060-Cytokine-cytokine receptor interaction (*P* = 6.87E-08), hsa04064-NF-kappa B signaling pathway (*P* = 2.38E-05), hsa04662-B cell receptor signaling pathway, and hsa04514-Cell adhesion molecules (CAMs) (*P* = 0.002).

### Protein-protein interaction (PPI) network construction and identification of hub genes

Approximately 149 nodes and 1,016 protein pairs were obtained when the combined weight score was set at more than 0.25 (Figure 3C-All). Analysis in the submodule revealed four modules with a score > 6 detected by MCODE (Figure 3C Cluster 1-4). After integrating the GO function, KEGG pathway enrichment and PPI analysis results, we identified several genes with a high degree and MCODE scores: *PRF1, GZMB, CD27, CD2, CCL5, CXCR5, CD8A, CCR7, IL2RB, IFNG, CD40LG, IL7R, CD226, LAT, CD6, PLCG1, CD22, CCR4, TNFRSF13B, PRKCQ, LTB, LTA, STAT4, TNFRSF13C*. Next, we needed to further verify these genes to determine their functional characteristics.

### Dataset validation

**Figure 4:**
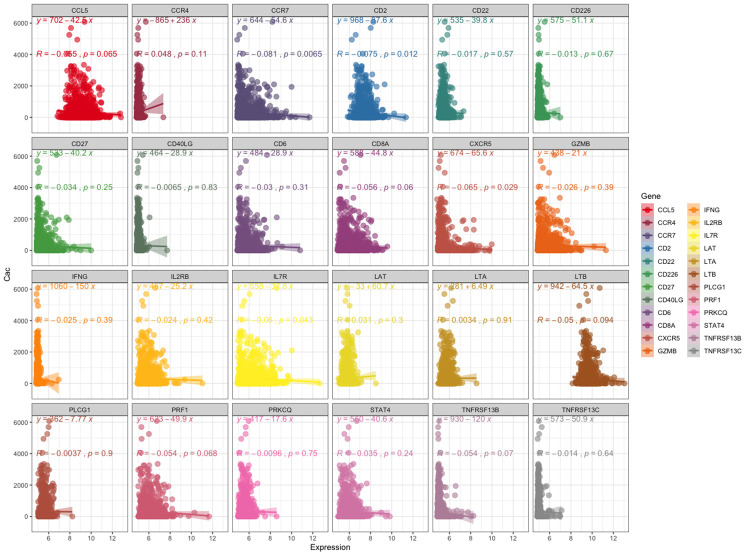
**Relationship between the expression level of these 24 genes and coronary artery calcification score.** The left panel shows the coronary artery calcification score (y-axis). The expression level of these 24 genes is shown on the x-axis.

**Figure 5:**
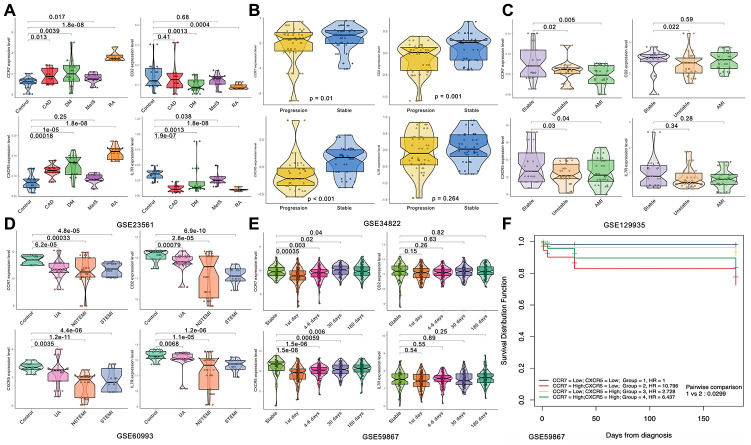
**Expression of the four hub genes in different datasets.** (**A**) GSE23561. (**B**) GSE34822. (**C**) GSE122935. (**D**) GSE60993. (**E**, **F**) GSE59867.

First, we analyzed the relationship between the expression level of these 24 genes and the coronary artery calcification score by linear correlation in GSE56045. As shown in Figure 4, only 4 gene expression levels were related to the coronary artery calcification score: *CCR7* (R = -0.081, *P* = 0.0065), *CD2* (R = -0.075, *P* = 0.0012), *CXCR5* (R = -0.065, *P* = 0.029) and *IL7R* (R = -0.06, *P* = 0.043). Next, we tested the expression level and function of the four genes in different datasets to confirm whether they were closely related to CAD. As shown in Figure 5A, the expression levels of *CCR7*, *CXCR5* and *IL7R* were different in the CAD and control group (*P* < 0.05–0.001), but no significant difference was found with *CD2* (*P* = 0.41). In GSE34822 (Figure 5B), the expression of *CCR7*, *CXCR5* and *CD2* was different in progressive and stable plaques, while that of *IL7R* was not significantly different (*P* < 0.05–0.001). Additionally, the gene expression level of progressive plaques was lower than that of stable plaques. Figure 5C analyzes GSE129935, which revealed that only *CCR7* and *CXCR5* had statistical significance in the comparison of stability and acute myocardial infarction (AMI), stability and instability (*P* < 0.05–0.01). Additionally, the subsequent decrease in expression led to the occurrence of plaque instability, which leads to AMI. GSE60993 (Figure 5D) also well verified the previous conclusion. From this dataset, the expression of these four genes in the healthy control group was statistically significant compared with unstable angina (UA), non-ST elevation myocardial infarction (NSTEMI) and ST elevation myocardial infarction (STEMI) (*P* < 0.001), and the healthy control group had a higher expression level. GSE59867 (Figure 5E) reflected the change in gene expression from stable to the first day to six months after AMI. From this dataset, we found that only *CCR7* and *CXCR5* expression was statistically significant (*P* < 0.05–0.001), the expression level was the lowest on the first day after AMI, and the gene level gradually increased with time. By dividing the expression of *CCR7* and *CXCR5* in GSE59867 by the mean value, expression greater than the mean value was defined as high expression, and the occurrence of heart failure was defined as the end event. We found that, when *CCR7* showed high expression and *CXCR5* showed low expression, the occurrence of heart failure was 10.796 times higher than that of *CCR7* and *CXCR5* (*P* < 0.05) (Figure 5F). Based on the above five datasets, we believe that *CCR7* and *CXCR5* play an important role in atherosclerosis and plaque vulnerability. Further verification of these data in a future investigation is warranted.

### Subject validation

**Figure 6:**
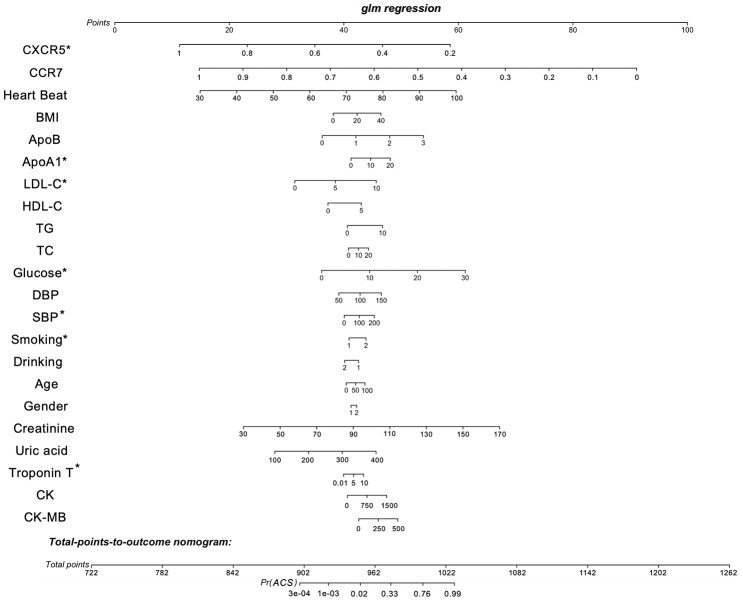
**Nomogram to estimate individual ACS probability.** Each predictor variable characteristic has a corresponding point value based on its position on the top point scale and contribution to the model. The probability of ACS for each subject is calculated by summing the points for each variable to obtain a total point value that corresponds to a probability of ACS from the scale presented on the bottom line. The variable data, including the relative expression of *CCR7* and *CXCR5*, gender, age, smoking, drinking, BMI, systolic blood pressure (SBP), diastolic blood pressure (DBP), serum glucose, TC, TG, high-density lipoprotein cholesterol (HDL-C), LDL-C, apolipoprotein (Apo)A1, ApoB and defined the sores as follows: smoking and/or drinking: yes = 2, no = 1; male = 1; female = 2. The predictive accuracy of the risk model was assessed by discrimination measured by C-statistic and calibration evaluated by Hosmer-Lemeshow χ^2^ statistic. The discriminatory ability of the model was quantified using the area under the receiver operating characteristic curve (AUC). The discrimination accuracy of the model was 0.841 (95% CI, 0.809–0.871). At an optimal cutoff value, the sensitivity and specificity were 64.0% and 90.9%, respectively. *P < 0.05.

To verify the exact function of *CCR7* and *CXCR5*, we collected the data of some patients hospitalized due to chest pain, measured the relative gene expression after collecting the peripheral blood, and compared the gene expression level according to the coronary angiography data. Table 1 shows the general situation of 1,528 patients with gender and age matching. We considered all the variable data, including the relative expression of *CCR7* and *CXCR5*, gender, age, smoking, drinking, BMI, systolic blood pressure (SBP), diastolic blood pressure (DBP), serum glucose, TC, TG, high-density lipoprotein cholesterol (HDL-C), LDL-C, apolipoprotein (Apo)A1 and ApoB, which were the best subset of risk factors to develop the acute coronary syndrome (ACS) risk score and risk model (nomogram) (Figure 6). We defined the sores as follows: smoking and/or drinking: yes = 2, no = 1; male = 1; female = 2. The nomogram had excellent discriminative power with a C-statistic and was well calibrated with the Hosmer-Lemeshow χ ^2^ statistic. The predicted probabilities of developing ACS ranged from 0.0004 to 99%. The discrimination accuracy of the model was 0.841 (95% CI, 0.809–0.871). At an optimal cutoff value, the sensitivity and specificity were 64.0% and 90.9%, respectively.

**Table 1:** Comparison of demographic, lifestyle characteristics and serum lipid levels among different groups.

**Parameter**	**Control**	**CAD**	**UA**	**NSTEMI**	**STEMI**
Number	312	320	304	290	302
Male/female	94/220	89/231	92/210	84/202	98/202
Age (years)^1^	55.32±8.31	54.16±9.05	55.59±8.54	56.43±9.83	55.94±9.17
Height (cm)	166.24±6.82	168.59±7.29	167.55±7.28	166.52±6.94	167.23±9.13
Weight (kg)	52.46±6.84	54.74±10.82	57.63±9.11^a^	57.92±8.77^a^	57.52±9.23^a^
Body mass index (kg/m^2^)	29.29±5.13	30.44±6.47^a^	30.67±7.12^a^	30.79±6.74^a^	30.77±6.09^a^
Waist circumference (cm)	74.49±6.74	73.55±9.48	76.03±8.15	76.24±7.17	76.15±6.27
Smoking status [*n* (%)]	81(26.0)	114(35.6) ^a^	142(46.7) ^a^	138(47.6) ^a^	144(47.6) ^a^
Alcohol consumption [*n* (%)]	75(24.0)	84(26.2)	70(23.0)	81(27.9) ^a^	71(23.5)
SBP (mmHg)	124.14±17.14	129.21±21.11^a^	147.15±23.96^c^	139.47±22.14^b^	103.45±17.16^c^
DBP (mmHg)	80.52±11.16	81.33±11.25	88.54±14.23^a^	84.43±11.21	68.54±12.15^c^
PP (mmHg)	49.64±14.13	51.42±13.59	51.66±15.24	50.84±15.22	50.22±14.21
Glucose (mmol/L)	5.91±1.83	6.13±2.22	7.79±2.43^b^	8.46±2.79^c^	8.69±2.78^c^
TC (mmol/L)	4.93±1.21	5.21±1.17^a^	5.63±1.12^a^	5.99±1.18^a^	5.83±1.43^a^
TG (mmol/L)^2^	1.49(0.51)	1.53(1.22)	1.52(1.21)	1.44(1.32)	1.51(1.26)
HDL-C (mmol/L)	1.52±0.44	1.32±0.26^a^	1.35±0.34^a^	1.44±0.28^a^	1.48±0.32
LDL-C (mmol/L)	2.86±0.81	3.23±0.74^a^	3.98±0.79^a^	3.79±0.88^a^	3.92±0.84^a^
ApoA1 (g/L)	1.24±0.24	1.17±0.22	1.18±0.26	1.18±0.26	1.14±0.28
ApoB (g/L)	0.84±0.19	0.82±0.32	0.81±0.31	0.94±0.31	0.88±0.28
ApoA1/ApoB	1.67±0.50	1.66±0.57	1.64±0.58	1.65±0.61	1.67±0.54
Heart rate (beats/minutes)	72.41±10.19	72.33±10.32	76.28±10.61^a^	76.43±9.31^a^	79.76±10.14^a^
Creatinine, (μmol/L)	72.34±12.22	71.36±11.34	74.53±11.62	76.55±10.23	76.58±12.74
Uric acid, (μmol/L)	283.83±76.19	286.89±74.32	279.88±81.31	285.91±81.31	283.86±75.28
Troponin T, (μg/L)	0.01±0.02	0.02±0.02	0.02±0.01	2.74±3.93^c^	2.86±6.28^c^
CK, (U/L)	88.84±45.28	87.84±48.31	91.88±50.33	1111.92±683.31^c^	1124.88±783.28^c^
CKMB, (U/L)	12.44±3.63	13.11±2.78	13.22±3.32	129.88±61.45^c^	132.76±59.17^c^

*CAD,* coronary artery disease; *UA*, unstable angina; *NSTEMI*, on-ST-segment elevation myocardial infarction; *STEMI*, ST-segment elevation myocardial infarction; *SBP*, systolic blood pressure; *DBP*, diastolic blood pressure; *PP*, pulse pressure; *TC*, total cholesterol; *TG*, triglyceride; *HDL-C*, high-density lipoprotein cholesterol; *LDL-C*, low-density lipoprotein cholesterol; *Apo*, Apolipoprotein. ^1^Mean ± SD determined by *t*-test.^2^Because of not normally distributed, the value of triglyceride was presented as median (interquartile range), the difference between the two groups was determined by the Wilcoxon-Mann-Whitney test. The P value was defined as the comparison of case and control groups. ^a^*P* < 0.05; ^b^*P* < 0.01; ^c^*P* < 0.001.

**Figure 7:**
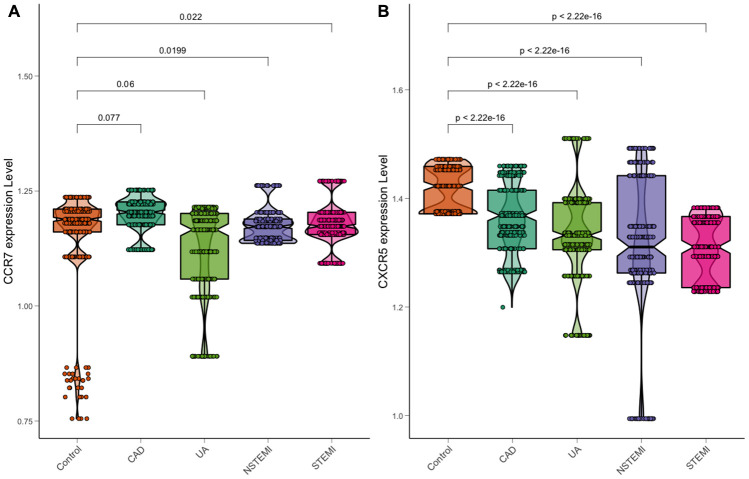
**Expression of CCR7 and CXCR5 in a chest pain patient.** (**A**) CCR7. (**B**) CXCR5.

After calculation, the relative expression of levels of *CXCR5*, ApoA1, LDL-C, serum glucose, smoking and Troponin T were significantly related to the risk of ACS, with statistical significance. The relative expression of peripheral blood RT–PCR showed that the expression of *CXCR5* was statistically significant in the comparison of cases (including UA, NSTEMI and STEMI) and controls, and the expression decreased gradually with the increase in plaque vulnerability (Figure 7), a finding that was also consistent with the results of previous multidatasets.

## DISCUSSION

Previously, the main cause of arteriosclerosis was considered the formation of atherosclerotic plaques caused by abnormal serum lipids. However, in recent years, with more extensive research, researchers have gradually realized that arteriosclerosis is a chronic inflammatory process with intense immune activity [9]. More evidence has also shown that LDL has synergistic effects with an immune inflammatory response, which promotes the formation of atherosclerosis. Considering blood lipid control alone, we have efficient tools to reduce LDL and the occurrence of cardiovascular adverse events (MACE). However, even if LDL is greatly reduced, MACE will still occur. This residual risk can be explained by the immune inflammatory response. Clear inflammatory interventions have now been shown to improve the prognosis of individuals treated with LDL-lowering drugs [10].

Previously, T and B cells were found in the detection of atherosclerotic plaques, which opened the door to the study of the autoimmune response leading to atherosclerosis [11]. Furthermore, the rate of APC-CD4 ^+^ T-helper cell interaction in atherosclerotic plaques of mice was increased, especially in hypercholesterolemia, leading to the secretion of pro-inflammatory cytokines [12]. Additionally, in lymph node biopsy, we found that auxiliary T cells gradually matured into antigen empirical effect/memory (T_EM_) and central memory (T_CM_) T cells, also observed in atherosclerotic plaques [13]. The sustained enhanced activation of T cells was accompanied by the expansion of lymph nodes in atherosclerotic ApoE ^- / -^ mice, as well as the local and systemic pro-inflammatory response, to further enhance the diet induced by hypercholesterolemia. These findings support the concept that specific antigens drive immune responses in the aorta and lymph nodes during atherosclerosis [14].

C-X-C motif chemokine receptor 5 (CXCR5), as a member of the CXC chemokine receptor family, encodes a multipass membrane protein. Recently, we identified characteristic CXCR5^+^-expressing follicular helper T cells (Tfh), a multifunctional helper T-cell subpopulation that helps B cells to differentiate into plasma membrane [15]. More evidence has shown that Tfh cells are also related to various inflammatory diseases, and an increased number of Tfh cells is found in CAD [16]. Through further experiments, we found that the circulating CD4 ^+^ CXCR5 ^+^ T cells in patients with CAD are rich in PD-1 ^+^ CCR7^-^ subsets, which can secrete IFN - γ, IL-17A and IL-21 in large quantities. Additionally, CD4 ^+^ CXCR5 ^+^ T cells in patients with CAD showed a stronger ability to stimulate the flow of B cells than that in healthy people. In coincubated B cells, the expression of IL-6 and IFN - γ also increased significantly, which may be the possible mechanism of CXCR5-induced arteriosclerosis [17]. Van der Vorst EPC et al found that CXCL13-CXCR5 chemokine axis plays an important role in the occurrence and development of atherosclerosis. The most likely reason is that the production and secretion of IgM protecting atherosclerosis by regulating the distribution pattern of B1 cells [18]. After further analysis of the expression quantitative feature loci of the gene expression data set, the G allele of rs77564610 and high expression of *CXCR5* in the whole blood were found to be closely related to the high risk of myocardial infarction [19]. However, common variations of *CXCR5* (50 kb) were found in the British biological bank cohort; the allele mutations of rs187248852 and rs73575424 are related to the pathogenesis of ischemic stroke and myocardial infarction, respectively [20]. This finding was consistent with ours.

This study possessed limitations. First, the patients enrolled to validate the relative expression in this study were from only one hospital, and the sample size was small. Whether there is a difference in patients from different areas and races is not known. Therefore, the validity of the findings should be tested in more prospective cohorts. Second, although we carried out many validations in other expression datasets and patients, the mechanism of *CXCR5* leading to atherosclerosis and plaque vulnerability remains unclear and needs further verification through more detailed in *vivo* and in *vitro* experiments.

In conclusion, we explored the molecular mechanism of ACS by performing weighted gene coexpression networks analysis. After functional and protein-protein interaction analysis, 24 genes were identified with a significant meaning. We further validated these genes in GSE23561, GSE34822, GSE59867, GSE60993 and GSE129935 datasets and found *CCR7* and *CXCR5* as the hub genes. Next, we validated these two genes in chest pain patients and found that *CXCR5* may play key role in atherosclerosis and plaque vulnerability; however, further confirmation is needed to validate the findings.

## MATERIALS AND METHODS

### Microarray data

All dataset analyses were performed using R software (version 3.60). We downloaded GSE56045 [21] microarray data from GEO (Gene Expression Omnibus, http://www.ncbi.nlm.nih.gov/geo/). These datasets were based on the platform of the GPL10558 Illumina HumanHT-12 V4.0 expression BeadChip. GSE56045 contains 1,202 peripheral blood samples, as well as clinical information, such as age, whole-blood cell count, and the coronary artery calcification plaque score. First, we added CEL files into the R software using the Affy package for transformation into an expression value matrix. The probe information was then transformed into gene names using the Bioconductor package. The mean value should be chosen when a gene had more than one probe [22]. The preprocessing process of the datasets (GSE23561, GSE34822, GSE59867 and GSE60993) [23–27] used for validation was the same as that for GSE56045. GSE129935 was performed by Fragments Per Kilobase per Million mapped reads (FPKM) and quantile normalized using the robust multiarray average (RMA) method. The probes were then annotated using Bioconductor in R. GSE129935 was also used for validation.

### Weighted gene coexpression network analysis

We conducted the analyses in strict accordance with the weighted gene coexpression network analysis process [28]. First, we chose the appropriate soft threshold power according to standard scale-free networks, with which adjacencies between all differential genes were calculated by a power function. Next, a topological overlap matrix (TOM) was derived from the adjacencies, and the corresponding dissimilarity (1-TOM) was counted. To complete the module recognition, we used the dynamic tree cutting method to cluster the genes in layers, using 1-TOM as the distance measure, a minimum size cutoff of 30, and a deepSplit value of cutting of 2. Next, we selected the highly similar modules by clustering and merged them, with the height line set as 0.4. To test the stability of each identified module, module preservation and quality statistics were computed using the module preservation function implemented in the WGCNA package [28]. Because the genes in the gray module cannot be attributed to any other module, all the genes in the gray module were removed.

### Interest module and hub gene

We selected the module with the highest correlation with clinical features and the genes in this module with the important biological functions. To identify the most biologically significant module, we used Pearson correlation analysis to evaluate the correlation between clinical features and gene modules. We selected the most relevant modules related to clinical features for further analysis and research. Gene Ontology (GO) and Kyoto Encyclopedia of Genes and Genomes (KEGG) pathway analyses were performed to detect potential mechanisms by which these module genes affect correlative clinical features. The thresholds for the P value and false discovery rate (FDR) were set as less than 0.01 and 0.05, respectively. These analyses were completed using clusterProfiler and DOSE package in R [29].

We describe the correlation between the gene expression profile and module eigengenes (Mes) defined as the module membership (MM). The gene significance (GS) can be defined as the absolute value of the correlation between external traits and the gene. The genes of interest in the modules with the highest MM and GS scores were selected for subsequent analysis. We defined the GS and MM scores as more than 0.2 and 0.6, respectively, for intramodular hub genes selected by external traits, and the P cutoff value is set at less than 0.05. The STRING database (https://string-db.org/, Version 11.0) [30] was employed to analyze the protein-protein interaction (PPI) network. We used Cytoscape software (version 3.80) to visualize and construct the gene-gene interaction network. Additionally, we used the Molecular Complex Detection (MCODE) app to screen the most notable clustering modules, with an MCODE score greater than 6 set as the threshold for further analysis [31].

### Hub gene validation and survival analysis

Our validation analysis was divided into two stages. In the first stage, we analyzed the function of the hub genes in different datasets; in the second stage, we verified the population. After collecting the patients admitted for chest pain, we extracted the peripheral blood and verified the expression of core genes. GSE23561, GSE34822, GSE59867, GSE60993 and GSE129935 were employed for validation. At first, we analyzed the correlation between the gene expression level and calcification score. Next, we compared the expression differences of hub genes among different groups and showed the results using the ggplot2 package in R. Subsequently, the “survival” package [32] in R was used to perform overall survival (heart failure) and disease-free survival analyses for all hub genes. The patients were divided into four groups (high vs. low) based on the hub gene expression level compared with the mean expression level of that hub gene. A Kaplan–Meier survival plot was also constructed. The ‘rms’ package was used for ACS prediction nomogram. The predictive accuracy of the risk model was assessed by discrimination measured by C-statistic and calibration evaluated by Hosmer-Lemeshow χ^2^ statistic. The discriminatory ability of the model was quantified using the area under the receiver operating characteristic curve (AUC). A 95% CI was calculated for each AUC. In general, an AUC > 0.75 was considered to be relatively good discrimination.

### Study population

In total, 1,528 patients were recruited from the inpatient department for a complaint of chest discomfort at the Liuzhou People's Hospital from 2018-3-1 to 2019-12-31 and had undergone coronary angiography. CAD, UA and AMI were diagnosed based on the Fourth Universal Definition of Myocardial Infarction (2018) [33]. Exclusion criteria included subjects with poor compliance, incomplete clinical data, contrast agent sensitivity and autoimmune diseases. Additionally, subjects with obvious surgical contraindications were excluded. Clinical data collection, biochemical measurements and diagnostic criteria were performed according to previous studies [34]. The study adhered to the Declaration of Helsinki of 1975 (http://www.wma.net/en/30publications/10policies/b3/) and its revision in 2008 and the Ethics Committee of Liuzhou People's Hospital agreed with the study design (No: Lunshen-2018-KY; Feb. 12, 2018). Informed consent was obtained from all subjects after receiving a full explanation of the study.

### RT–qPCR and statistical analysis

The procedures of blood sample collection, RNA isolation, reverse transcription cDNA and RT–qPCR are the same as those in our previous studies and were carried out in strict accordance with the product instructions and laboratory operating procedures [35]. The specific divergent primers were designed to amplify the transcripts and are shown in Supplementary Table 3. The statistical software package SPSS 22.0 (SPSS Inc., Chicago, IL, USA) and R software (version 3.6.0) were used for statistical analysis. Quantitative variables were expressed as means ± standard deviation (TG levels were shown as medians and interquartile ranges and were analyzed by the Wilcoxon–Mann–Whitney test because they were not a normal distribution). Chi-square analysis was used to assess the difference in the percentages between the groups. All tests were two-sided, and *P* < 0.05 was considered statistically significant.

### Ethics approval

This analysis of publicly available data does not require ethical approval.

## Supplementary Material

Supplementary Table 1

Supplementary Table 2

Supplementary Table 3
