# Supplementary material for: Weighted gene coexpression network analysis identifies the key role associated with acute coronary syndrome
Source: Aging (Albany NY). 2020 Oct 14;12(19):19440–54. doi: 10.18632/aging.103859 (PMC7732301; doi:10.18632/aging.103859)
Supplement: Supplementary Table 3 [file aging-12-103859-s003..pdf]

SUPPLEMENTARY TABLE

Supplementary Table 3. The primer of CXCR5 and CCR7.

Gene	Forward primer	Reverse primer
CCR7	GGGCCCAGCAGGAACTTATT	GTCAGAGCGGGCTTTCTCTA
CXCR5	ACATCCTTTGCCAGAGTCCG	AACTCTTAAAGGGCGGGAGC
